# Reaction Wood Anatomical Traits and Hormonal Profiles in Poplar Bent Stem and Root

**DOI:** 10.3389/fpls.2020.590985

**Published:** 2020-12-07

**Authors:** Elena De Zio, Antonio Montagnoli, Michal Karady, Mattia Terzaghi, Gabriella Sferra, Ioanna Antoniadi, Gabriella S. Scippa, Karin Ljung, Donato Chiatante, Dalila Trupiano

**Affiliations:** ^1^Department of Biosciences and Territory, University of Molise, Pesche, Italy; ^2^Department of Biotechnology and Life Science, University of Insubria, Varese, Italy; ^3^Umeå Plant Science Centre, Department of Forest Genetics and Plant Physiology, Swedish University of Agricultural Sciences, Umeå, Sweden; ^4^Laboratory of Growth Regulators, Institute of Experimental Botany of the Czech Academy of Sciences and Faculty of Science of Palacký University, Olomouc, Czechia; ^5^Department of Chemistry and Biology ‘A. Zambelli’, University of Salerno, Fisciano, Italy

**Keywords:** bending stress, auxins, cytokinins, metabolite profiling, UHPLC-MS/MS

## Abstract

Reaction wood (RW) formation is an innate physiological response of woody plants to counteract mechanical constraints in nature, reinforce structure and redirect growth toward the vertical direction. Differences and/or similarities between stem and root response to mechanical constraints remain almost unknown especially in relation to phytohormones distribution and RW characteristics. Thus, *Populus nigra* stem and root subjected to static non-destructive mid-term bending treatment were analyzed. The distribution of tension and compression forces was firstly modeled along the main bent stem and root axis; then, anatomical features, chemical composition, and a complete auxin and cytokinin metabolite profiles of the stretched convex and compressed concave side of three different bent stem and root sectors were analyzed. The results showed that in bent stems RW was produced on the upper stretched convex side whereas in bent roots it was produced on the lower compressed concave side. Anatomical features and chemical analysis showed that bent stem RW was characterized by a low number of vessel, poor lignification, and high carbohydrate, and thus gelatinous layer in fiber cell wall. Conversely, in bent root, RW was characterized by high vessel number and area, without any significant variation in carbohydrate and lignin content. An antagonistic interaction of auxins and different cytokinin forms/conjugates seems to regulate critical aspects of RW formation/development in stem and root to facilitate upward/downward organ bending. The observed differences between the response stem and root to bending highlight how hormonal signaling is highly organ-dependent.

## Introduction

Mechanical stimuli (e.g., rain, wind, gravity, soil impedance, wounding, and bending) can considerably influence plant growth and development. Plants have developed sensory mechanisms to detect mechanical perturbations and to induce a suite of responses (anatomical, physiological, biochemical, biophysical, and molecular) collectively termed “thigmomorphogenesis” (Jaffe and Forbes, 1993; Braam, 2005; Dumroese et al., 2019). Thigmomorphogenesis can be considered as an adaptive response allowing individual plants to mitigate mechanical stress that occurs in their natural environment.

Over the past decades, different typologies of mechanical stress (in term of duration, targeted plant organ, or plant developmental stage) have been used to investigate thigmomorphogenic response in a wide range of plant species. Meanwhile, this phenomenon was documented in all types of plants, indicating its wide conservation (Jaffe and Forbes, 1993). Several short-time scale studies indicate a transient cessation of plant growth upon a mechanical stimulus. Conversely, continued long-term stem flexing led to an increase in root/shoot dry weight ratios, without affecting root length or total biomass, compared to unflexed plants (Garner and Björkman, 1999).

Beyond the macroscopically visible morphogenic changes, mechanical stress also affects wood mechanical properties (Chiatante et al., 2003; Montagnoli et al., 2020). In general, vascular cambium of trees growing in a windy environment produces a specific wood called “flexure wood” (FW) by increasing secondary xylem production and decreasing the elastic modulus in comparison to normal wood (NW) formed in absence of stimulus. In windy natural settings, bending occurs in a non-symmetric way, with the leeward portion of the stem experiencing more compression stress and the windward part more tension stress. Hence, what is really FW? The anatomy and specific functions of FW are poorly understood, but it needs to function in both compression and tension, due to the alternating sway, to reduce the risk of mechanical failure of the stem (Telewski, 1989; Kern et al., 2005; Koehler and Telewski, 2006).

Gymnosperms and angiosperms have evolved another strategy to counteract the bending constraint with secondary growth, which implies the asymmetrical formation of the so-called reaction wood (RW). This secondary xylem tissue is anatomically different from the NW and from the opposite wood (OW), commonly located on the opposite side of RW and characterized by properties between NW and RW (Timell, 1986). It, contrary to FW, does not form in response to swaying but due to displacement of the stem or root for times much longer than the presentation time (minimal time to produce a response) of gravitropism (Gardiner et al., 2016).

In gymnosperms, the RW is referred to as compression wood (CW) and develops on the lower side of leaning stems or branches, whereas, in dicotyledonous angiosperms, it forms on the upper side and is called tension wood (TW; Du and Yamamoto, 2007).

TW (more contractile) is often characterized by the formation of a gelatinous layer within the fiber cell wall (G-fibers) thought to be poorly lignified, and mainly composed of cellulose with the potential additions of arabinogalactan and xyloglucan differing from the normal fiber cell wall (Nishikubo et al., 2007; Bowling and Vaughn, 2008). Conversely, the typical CW contains more lignin, and has a flatter microfibril angle and lower cellulose content than NW and TW, being thus characterized by a high rigidity (Pilate et al., 2004). Stems and roots subjected to similar mechanical forces may develop extremely different RW. Indeed, in poplar, bending induces TW formation on the “upper” convex (tension) side of the stem or branch (Du and Yamamoto, 2007). Conversely, in poplar root, De Zio et al. (2016) for the first time observed that a CW similar to gymnosperm stems was formed in the “lower” concave (compression) side after bending.

Authors observed many similarities between FW and RW (CW or TW) formed in both gymnosperms and angiosperms (Butterfield and Li, 2000; Kern et al., 2005). However, the effect of elementary stresses (swaying, compression, and tension loadings) on wood anatomy, structure, and function is not completely known.

In the last 2 decades, computer modeling has helped to understand how mechanical forces are loaded on plant organs showing patterns coherent with direct measurement and able to explain the induced alterations (Danjon et al., 2005; Yang et al., 2014, 2016; Montagnoli et al., 2020). It is evident that tension and compression loadings induce complex signal transduction pathways that involve other factors such as phytohormones, which are still poorly understood, especially at the root level (Du and Yamamoto, 2007).

Functional role of auxin in plant response to mechanical stress has been an active area of research on woody stems. However, findings on the relationship between endogenous auxin levels and the formation of CW or TW are still scarce and sometimes contradictory, thus remaining to be elucidated (Du and Yamamoto, 2007). Hellgren et al. (2004) found that the formation of TW and CW in poplar and pine bent stems, respectively, are not mediated by changes in the indole-3-acetic acid (IAA) level in the cambial tissues. On the contrary, Funada et al. (1990) and Du et al. (2004) detected higher amount of endogenous IAA at the side of the cambial region forming CW. In line with these findings, we also found that compression forces induce wood formation with the intermediation of high IAA levels (De Zio et al., 2016), which could act as a spatial regulator of cambial activity, enhancing cell division rate and conferring key positional information to the cells of the cambial zone surrounding tissues for differentiation/RW initiation. Unlike IAA, the role of cytokinins (CKs) in RW formation has seldom been investigated (Little and Pharis, 1995; Mellerowicz et al., 2001). In *planta*, CKs occur in four principal forms: (i) the nucleotides (iPRMP, *t*ZR5MP, *c*ZRMP, and DHZMP) which are produced by *de novo* biosynthesis and then converted to other derivatives; (ii) the free bases (iP, *t*Z, *c*Z, and DHZ) which are considered to have the highest activity together with (iii) the ribosides (iPR, *t*ZR, *c*ZR, and DHZR) which are also preferably transport; and (iv) the glucosides which are temporary (*O*-glycosylation) or permanent (*N*-glycosylation) storage of inactivated forms (Spíchal, 2012). The *O*-glucoside, together with the ribosides, represents the form less susceptible to degradation by cytokinin oxidase and readily converted to the free base forms (Kieber and Schaller, 2014). Among the free bases, *c*Z is believed to have a lower activity compared to *t*Z or iP, which are generally considered the most biologically active CKs positively controlling overall plant growth (Schafer et al., 2015).

Despite the well-established functions of CKs in cell division, tracheid differentiation, and lignin biosynthesis (Aloni et al., 2006), no direct relation between CKs and RW formation was found in bent stem. Conversely, in the bent root, we found that specific and distinct CK types/forms in the vascular tissue control RW formation toward the compressed side (De Zio et al., 2016, 2019). Although evidence on the involvement of plant hormones has been provided (Trupiano et al., 2012b; Miyashima et al., 2013; Ursache et al., 2013; De Zio et al., 2016), differences and/or similarities existing between poplar bent roots and stems, especially in relation to the different intensity of tension and compression forces perception, remain unknown.

In the present study, firstly we assume that static bending in woody root and stem would induce an asymmetrical distribution of mechanical forces along the different stretched and compressed sides and sectors (bent sector and above/below the bent sector). Secondly, the different forces perceived by the two bent organs would produce also an asymmetrical phytohormones accumulation, able to trigger the formation of RW with differentiated characteristics in anatomical traits and chemical composition. To test our hypotheses, the woody *Populus nigra* plant responses to static, non-destructive, mid-term bending treatment was investigated along the stem and root axes, in stretched and compressed sides, by (i) developing a theoretical model to assess the type and magnitude of mechanical forces distribution, and (ii) analyzing anatomical features, chemical composition, and IAA and CKs metabolites profiling.

## Materials and Methods

### Plant Material and Bending Conditions

Static non-destructive mid-term (5 months) bending constraint was applied to 2 year-old *P. nigra* plants (*n* = 5). The root bending simulation was performed by tying taproots around right angle curved steel nets, as previously described in De Zio et al. (2016). The same bending angle (~90°) and similar supporters were used to impose the stress at stem level of other five poplar plants (Supporting information 1). All plants were grown for 5 months in a growth chamber at 22°C and 60–70% humidity with a 16 h photoperiod simulated by LED lights (*𝜆*_420_–*𝜆*_740_) and a photosynthetically active radiation of 350 μmol m^−2^ s^−1^ (Light Meter HD2302.0, Delta Ohm, Caselle di Selvazzano, Italy), ensuring controlled conditions.

At the end of the 5-months of bending treatment, a detailed spatial sampling and analysis was performed. Firstly, from both root and stem were taken three longitudinal sectors, each 5 cm long: (1) above bending sector (ABS), corresponding to the region just above the bending zone; (2) bending sector (BS), representing the point of maximum bending; and (3) below bending sector (BBS), corresponding to the region just below the bending zone. In the case of bent roots, ABS was localized at 12–17 cm distant from the root collar, BS at 17–22 cm, and BBS at 22–27 cm. Distances were inverted in the case of stem (ABS at 22–27 cm, BS at 17–22 cm, and BBS at 12–17 cm). Secondly, each region (ABS, BS, and BBS) was further divided lengthwise into two parts to collect both the convex (CX) and concave side (CE; Supplementary Figure S1). Immediately upon collection, the samples were frozen in liquid nitrogen and stored at −80°C for chemical analysis and hormone profiling or fixed in formalin-acetic acid-alcohol (FAA, 5:5:90) for anatomical investigations.

### Models of Bending Forces Distribution

Mechanical forces distribution along the bent stem and root models was performed by Mecway finite element analysis package (version 9.0; Mecway, 2014), considering the diameter and wood mechanical property of the two organs. At the beginning (t_i_) and at the end (t_f_) of the bending treatment, stem and root diameters of 15 plants were measured by using ImageJ 1.41o software (Wayne Rasbanb, National Institute of Health, United States). As already described in Fourcaud et al. (2008) and in Montagnoli et al. (2020), plant material was considered isotropic, uniform, and elastoplastic with a density of 1,000 kg/m^3^, with Young’s modulus of 5 GPa and a Poisson’s ratio of 0.3. Bending stress was applied to a total axis length of 15 cm through a forced displacement at the narrow end of the stem or root. Tension and compression forces were calculated considering the mesh average characterized by a total of 15 sections (1 cm each). Plant diameters from five adjacent sections were used to compose a specific organ sector (ABS, BS, and BBS) and, comprehensively, were used to construct a 2D section of the organ. A 3D model was derived by revolving the 2D section on this longitudinal axis.

### Anatomical Investigations

Each bent root and stem sector (ABS, BS, and BBS) fixed in FAA was dehydrated using a graded ethanol series (10, 30, 50, 70, 95, and 100%) and embedded using the Technovit 7,100 resin system (Heraeus Kulzer, Wehrheim, Germany) based on 2-hydroxyethyl-methacrylate.

Samples were sectioned into cross-sections (12 μm thick) using a sliding microtome. Finally, sections were stained in Toluidine Blue O (Parker et al., 1982) for 1 min. Sections were photographed using an Olympus BX63 light microscope equipped with an Olympus DP72 camera. Images were analyzed by ImageJ 1.41o software (Wayne Rasbanb, National Institute of Health, United States). In order to define the convex and the concave sides precisely, a 45°C rotated graphic crosswise object was applied, having the center of the primary xylem stele as the anchor point. In the convex and concave sides of three bent stem and root sectors, the following parameters were measured: cambial cell number (CCN), cambial zone thickness (CZT; μm), vessel wall thickness (VWT; μm), fiber wall thickness (FWT; μm), relative xylem thickness (RXT%), relative phloem thickness (RPT%), relative vessel area (RVA), relative vessel number (RVN), specific vessel area (SVA), and specific vessel number (SVN). Measurements were carried out in the areas formed after the application of bending (Supplementary Figure S1) following calculation reported in De Zio et al. (2016).

### Lignin and Carbohydrate Determination

Pyrolysis-gas chromatography/mass spectrometry (Py-GC/MS) was used to analyze lignin and carbohydrate content of bent woody root and stem samples. For the analysis, 50 μg (±10 μg) of ball-milled (MM400, Retsch, Germany) wood powder was applied to a pyrolyzer equipped with an auto sampler (PY-2020iD and AS-1020E, Frontier Lab, Japan) connected to a GC/MS (Agilent 7890A/5975C; Agilent Technologies AB, Sweden). The pyrolysate was separated and analyzed according to Gerber et al. (2012). After chromatograms processing, peaks were automatically classified and integrated. Classification defined peaks into syringyl (S), guaiacyl (G), and p-hydroxyphenyl (H)-type lignin, carbohydrates (C), according to the highest abundant *m/z* channel.

### Phytohormones Measurement

Indole-3-acetic acid (IAA) metabolites and CKs were extracted from 20 mg of fresh weight stem/root material (each pooled from five poplar plants), as described in De Zio et al. (2019). Vacuum-dried auxin and cytokinin fractions were dissolved in 10% methanol and stored at −20°C until ultra-high-performance liquid chromatography/tandem mass spectrometry (UHPLC-MS/MS) analysis.

Separation and determination of compounds was performed using a 1,290 Infinity LC system and 6,490 Triple Quadrupole LC/MS system (Agilent Technologies). Auxins mass analysis was done according to Novák et al. (2012), while CKs mass analysis was carried out in accordance with Novák et al. (2008). IAA metabolites were expressed as pg. mg^−1^ of dry weight, while CKs as pmol g^−1^ of dry weight. MassHunter software (version B.05.02; Agilent Technologies) was used to determine the concentrations of all examined compounds, using stable isotope dilution method.

### Statistical Analysis

When needed, variables were square root or log transformed, to ensure normal distributions and equal variances for the use of parametric statistics. As anatomical data did not follow the normal distribution, nonparametric statistics were applied. The Kruskal-Wallis multiple-comparison test was used to compare anatomical measurements for the two plant organs (stem and root), sectors (ABS, BS, and BBS), and sides (CX and CE). The Mann-Whitney U-test was used for pairwise comparison of anatomical measurements among root sectors for each of the two sides and to compare convex and concave sides within each sector. A 95% significance level was applied to analysis with nonparametric methods. For phytohormones analysis, a one-way ANOVA was used to compare different plant organs (stem and root), sectors (ABS, BS, and BBS), and sides (CX and CE). *Post-hoc* LSD-tests were conducted to detect overall differences between convex and concave sides for each sector of each plant organ. Analyses were applied on a 95% significance level. All statistical analysis was carried out using statistical software package SPSS 17.0 (SPSS Inc., Chicago IL, United States).

Finally, in order to investigate variance among different analytical dataset obtained from each sector of both the organs, we performed a principal components analysis (PCA). The main anatomical parameters (CCN, RXT, and RPT) and phytohormones (IAA and CK free forms – *c*Z, *c*ZR, *t*Z, *t*ZR, iP, iPR, and DHZ), together with total lignin amount, were analyzed by using FactoMineR package in R (Husson et al., 2014; R Core Team, 2020).

## Results

### Mechanical Force Distribution Model

**Figure 1:**
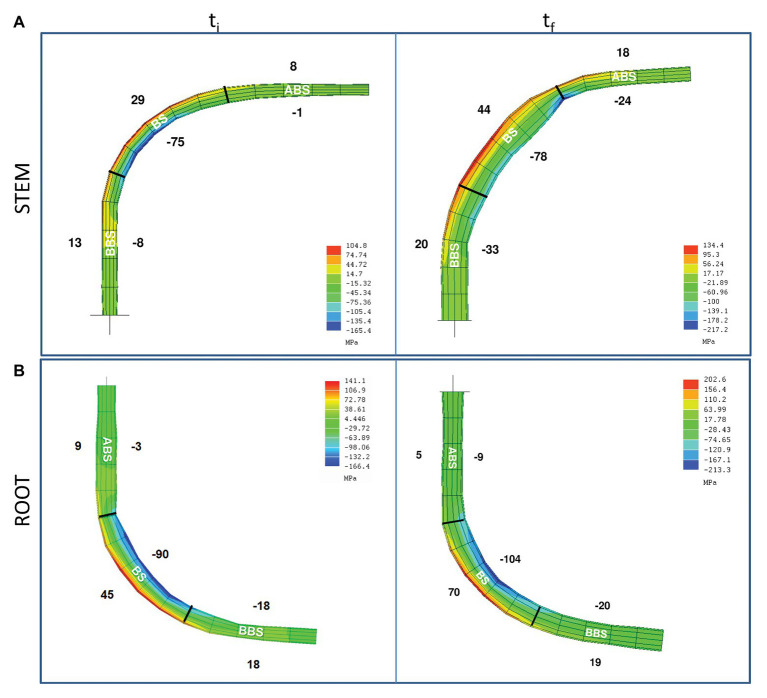
Model of the mechanical forces distribution along *P. nigra* bent stem and root. Mechanical forces distribution along the stem **(A)** and root **(B)** main axis, at the beginning (t_i_) and the end (t_f_) of bending treatment. Average value (MPa) of the mechanical force magnitudes are indicated on the corresponded concave (negative values – compression) and convex (positive values – tension) sides of three bent sectors (ABS, BS, and BBS). ABS, above bending sector; BS, bending sector; BBS, below bending sector.

The modeling of mechanical force distribution along bent stem and root axis showed maximum values of compression and tension forces in BS compared to ABS and BBS sectors of both organs (Figure 1). In general, the magnitude was higher for compression forces, rather than tension; both of them were greatest in the BS and were dissipated away from it, showing higher values in BBS than ABS.

All forces increased from the initial (t_i_) to the final (t_f_) phase of the bending application with different intensities depending on the sector analyzed (Figure 1). In stem, from t_i_ to t_f_, tension forces increased in the ABS and BS, remaining almost unchanged in the BBS, while compression forces greatly increased in the ABS and BBS and were similar in the BS.

In root, tension forces strongly increased in the BS, whereas remained almost unchanged in the ABS and BBS; compression forces increased slightly in the BS, 3-fold in the ABS, and unchanged in the BBS (Figure 1).

### Anatomical Traits

**Figure 2:**
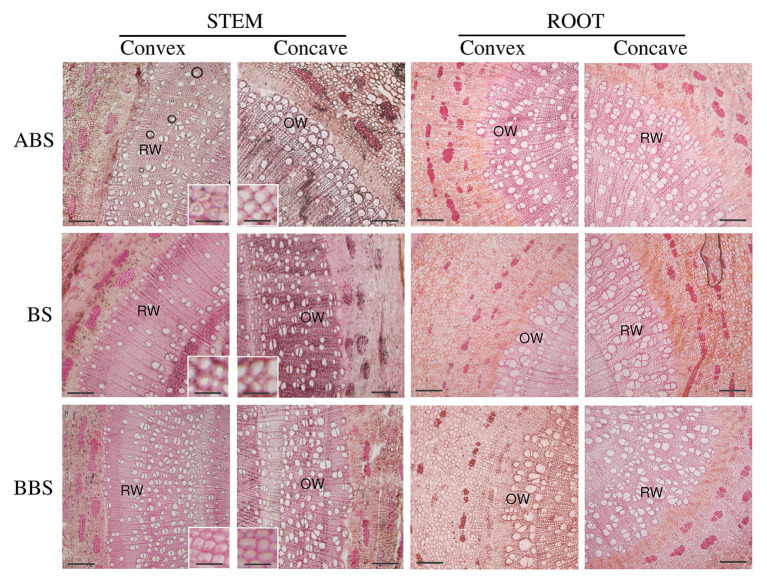
Anatomical bent stem and root cross-sections. Cross-sections of the convex and concave sides of three bent stem and root sectors (ABS, BS, and BBS) stained with Toluidine Blue O. Scale bar = 20 μm. Magnification shows secondary wood fiber cell wall characteristics. Scale bar = 2 μm.

Cross-sectional anatomical analysis of the bent stems showed that CCN did not differ among sectors, and for the ABS was lower in the convex side than in the respective concave side. No differences were detected among sectors and sides for the thickness of CZT (Table 1). The RXT did not differ among the three sectors while was larger in the convex side of both ABS and BS than the respective concave sides (Figure 2 and Table 1). The specific vessel number (SVN) and vessel wall thickness (VWT) did not differ among sectors, but it was higher in the concave side of ABS than in the respective convex side. The relative phloem thickness (RPT) did not differ between the two sides within each specific sector but showed the highest and the lowest values in the concave BBS and ABS, respectively, and intermediate value in the BS (Table 1). The fibers wall thickness (FWT) did not differ between the two sides within each specific sector and showed a lower value in convex BS only compared to BBS. In addition, fiber cell walls of convex ABS and BS were characterized by the formation of gelatinous layer (G-layer; magnification in Figure 2). Specific vessel area (SVA) did not differ among sides within each specific sector but showed the highest and the lowest value in the both sides of BBS and ABS, respectively (Table 1).

**Table 1:** Stem and root anatomical traits.

		STEM			ROOT		
		Sector			Sector		
Anatomical parameter	Side	ABS	BS	BBS	ABS	BS	BBS
CCN	CX	**3.42** ± 0.37^*^	4.72 ± 0.52	4.29 ± 0.53	4.31 ± 0.29	**4.46** ± 0.29^*^	**4.42** ± 0.23^*^
	CE	**4.65** ± 0.32^*^	4.56 ± 0.67	4.08 ± 0.35	4.62 ± 0.16	**5.62** ± 0.40^*^	**5.65** ± 0.47^*^
CZT	CX	14.04 ± 1.69	18.65 ± 3.00	17.27 ± 1.93	24.28 ± 1.78	**25.18** ± 2.13^*^	**22.37** ± 1.63^*^
	CE	18.17 ± 2.81	21.82 ± 3.20	18.01 ± 1.48	**26.87** ± **1.52**^b^	**32.72** ± **1.88**^a,*^	**35.45** ± **2.70**^a,*^
RXT	CX	**10.44** ± 1.60^*^	**12.26** ± 1.28^*^	10.13 ± 2.81	4.94 ± 1.09	**5.72** ± 0.92^*^	**6.31** ± 1.06^*^
	CE	**6.47** ± 2.09^*^	**5.70** ± 1.21^*^	7.69 ± 1.00	8.06 ± 0.70	**9.67** ± 0.60^*^	**10.23** ± 1.14^*^
SVN	CX	**275** ± 51^*^	303 ± 98	458 ± 54	**348** ± **11**^b^	**420** ± **28**^a^	**396** ± **74**^a,b^
	CE	**434** ± 13^*^	258 ± 100	552 ± 48	291 ± 51	332 ± 31	377 ± 41
VWT	CX	**1.15** ± 0.13^*^	1.29 ± 0.06	1.36 ± 0.07	1.65 ± 0.12	1.73 ± 0.13	1.76 ± 0.12
	CE	**1.47** ± 0.15^*^	1.28 ± 0.11	1.45 ± 0.12	**1.74** ± **0.11**^b^	**2.03** ± **0.05**^a^	**2.04** ± **0.11**^a,b^
RPT	CX	8.24 ± 1.19	9.43 ± 0.36	9.31 ± 0.67	16.14 ± 1.36	**17.07** ± 1.34^*^	**18.88** ± 0.80^*^
	CE	**6.90** ± **1.69**^b^	**7.79** ± **0.25**^a,b^	**9.10** ± **0.86**^a^	**16.75** ± **0.88**^b^	**21.66** ± **1.06**^a,*^	**21.26** ± **0.77**^a,*^
FWT	CX	**0.71** ± **0.07**^a,b^	**0.63** ± **0.04**^b^	**0.79** ± **0.04**^a^	0.88 ± 0.04	0.80 ± 0.04	0.84 ± 0.04
	CE	0.77 ± 0.05	0.66 ± 0.06	0.76 ± 0.03	0.97 ± 0.05	0.88 ± 0.05	0.86 ± 0.06
SVA	CX	**5.59** ± **0.91**^b^	**7.82** ± **3.00**^a,b^	**14.30** ± **2.39**^a^	30.8 ± 2.9	34.8 ± 1.7	33.2 ± 5.1
	CE	**9.17** ± **1.21**^b^	**8.10** ± **3.07**^b^	**22.01** ± **2.43**^a^	**25.9** ± **3.6**^b^	**31.5** ± **2.3**^a,b^	**38.0** ± **2.7**^a^

Anatomical parameters were analyzed for stem and root in the convex (CX) and concave (CE) sides of three bent stem and root sectors (ABS, BS, and BBS). Reported values are the mean of five replicates (±SE). Bold value indicates significant differences (*p* < 0.05) between the three bent sectors of the same side (different letters) and between convex and concave sides of the same sector (asterisk). ABS, above bending sector; BS, bending sector; BBS, below bending sector; CCN, cambial cell number; CZT, cambial zone thickness; RXT, relative xylem thickness; SVN, specific vessel number; VWT, vessel wall thickness; RPT, relative phloem thickness; FWT, fiber wall thickness; and SVA, specific vessel area.

Cross-sectional anatomical analysis of the root showed that the CCN and RXT did not show any significant difference among sectors, whereas the CZT and RPT, in the concave side, were higher in both BS and BBS than in the ABS (Figure 2 and Table 1). However, all previous parameters (CCN, CZT, RXT, and RPT) measured in the concave side of both BS and BBS were higher than in the respective convex side (Figure 2 and Table 1). SVN and VWT did not differ within sectors and showed the lowest and highest values only on the convex side of the ABS and BS, respectively, with intermediate value in the BBS (Table 1). FWT did not show any significant difference among sides and sectors, whereas the SVA differed only between different sectors on the concave sides showing low, middle, and high value in ABS, BS, and BBS, respectively (Table 1).

### Carbohydrate and Lignin Content

**Figure 3:**
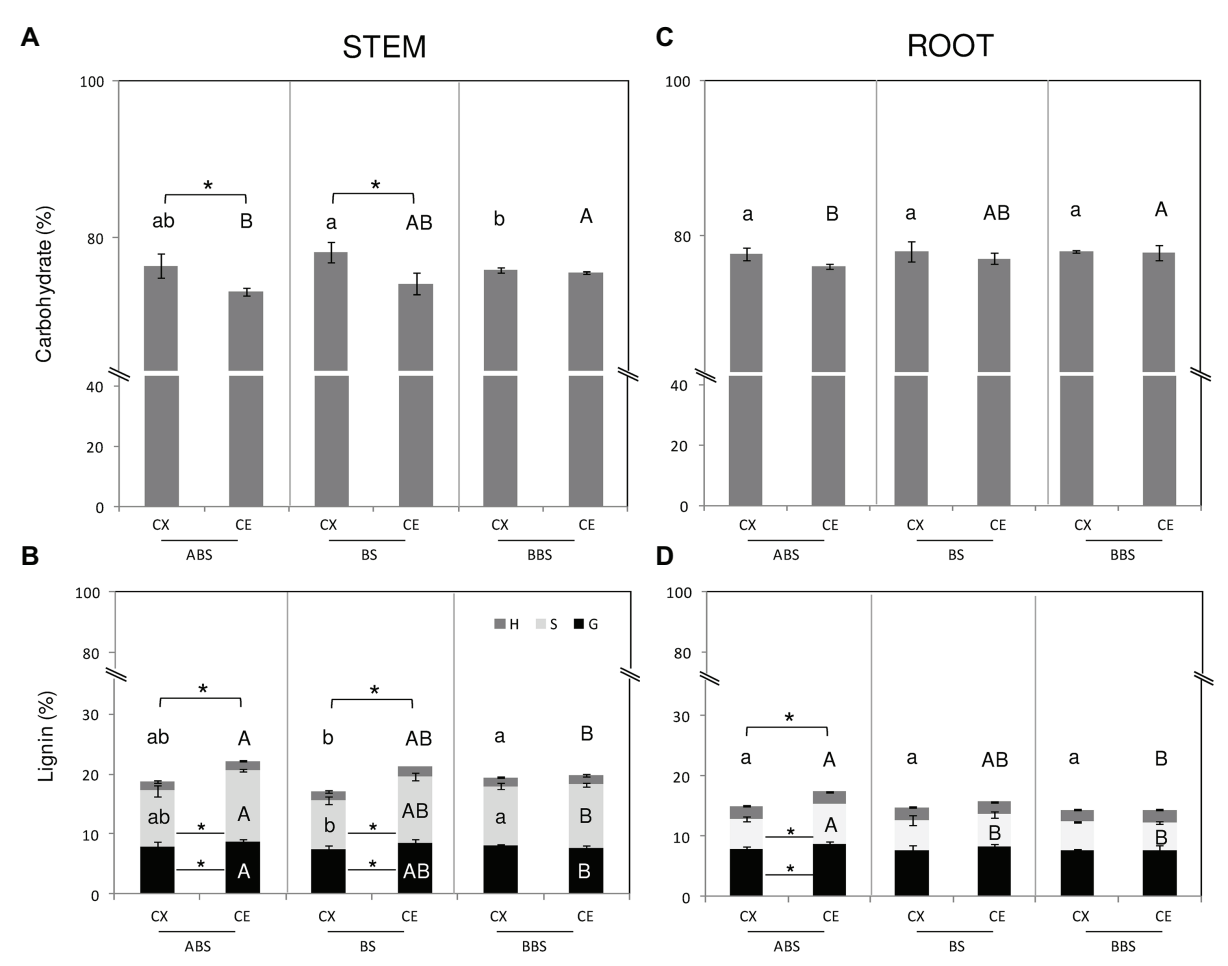
Carbohydrate and lignin content of *P. nigra* stem and root. Total carbohydrate and lignin content from CX and CE sides of ABS, BS, and BBS are indicated in the **(A)** and **(B)** for the bent stem and in the **(C)** and **(D)** for the bent root. Percentage value represents the mean of five independent samples ±SD analyzed by Py-GC/MS. Significant differences (*post-hoc* LSD-tests, *p* < 0.05) among the CX sectors are indicated by lower letters whereas those among CE sectors by capital letter. Significant differences (*post-hoc* LSD-tests, *p* < 0.05) between sides of the same sector are indicated by asterisk. Differences in total lignin amount are indicated above the histograms while differences in Syringyl- (S-), Guaiacyl- (G-), and p-Hydroxyphenyl- (H-) types lignin are indicated inside the histograms. ABS, above bending sector; BS, bending sector; BBS, below bending sector; CX, convex side; CE, concave side.

The comparison of Py-GC/MS data among different bent stem sectors showed that carbohydrates were higher in the convex side of BS than BBS and unchanged in ABS, whereas in the concave sides, they were higher in ABS than BBS and unchanged in BS (Figure 3A). Total lignin in the convex sides was lower in BS than BBS, due to a lowest S-type lignin, and unchanged in ABS (Figure 3B). No variations were observed along different sectors of concave sides. Furthermore, within sectors, convex ABS and BS were characterized by higher carbohydrates amount and lower total lignin content compared to the opposite concave sides (Figures 3A,B); variations resulted mainly due to a lower amount in G- and S-type lignin, whereas H-types remained unchanged (Figure 3B).

In case of bent root, results revealed that in the concave side, carbohydrates were lower in ABS than BBS and unchanged compared to BS (Figure 3C) whereas in the convex side, no significant change was found. Total lignin showed an opposite trend in the concave sides, being higher in ABS than BBS and unchanged compared to BS, while it was completely unvaried in the convex side (Figure 3D). The change in lignin content was mainly due to the alternation in S-lignin type amount. Convex and concave sides of all three sectors showed similar carbohydrate content (Figure 3C). Lignin content increased only in the concave ABS compared to the opposite convex side and unchanged in the two sides of BS and BBS. The increase in total lignin amount in the concave ABS was due to an increase of G- and S-type lignin (Figure 3D).

### Phytohormones Measurement

**Figure 4:**
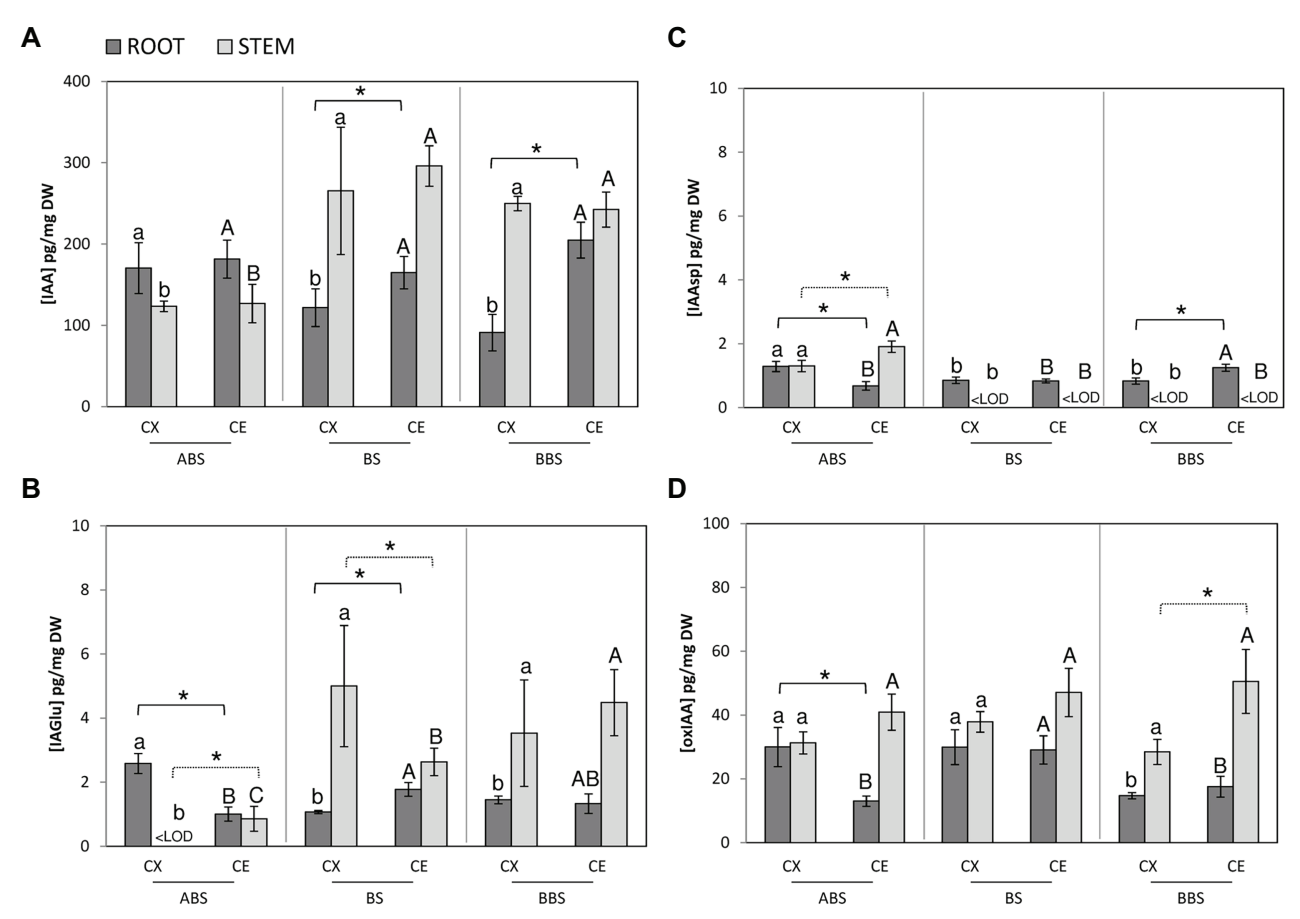
Auxin metabolites profiling in different bent sides and sectors of *P. nigra* stem and root. Concentrations of IAA **(A)**, IAGlu **(B)**, IAAsp **(C)**, and oxIAA **(D)** were analyzed by UHPLC-MS/MS. The values are expressed in pg. mg^−1^ of dry weight (DW). Data represent the mean of three independent extractions ±SD. All significant differences (*post-hoc* LSD-tests, *p* < 0.05) between the three bent sectors in convex and concave sides are indicated by minuscule and capital letter, respectively. Significant differences (*post-hoc* LSD-tests, *p* < 0.05) between sides of the same sector are indicated by asterisk and continuous line for root or dashed line for stem. ABS, above bending sector; BS, bending sector; BBS, below bending sector; CX, convex side; CE, concave side.

**Figure 5:**
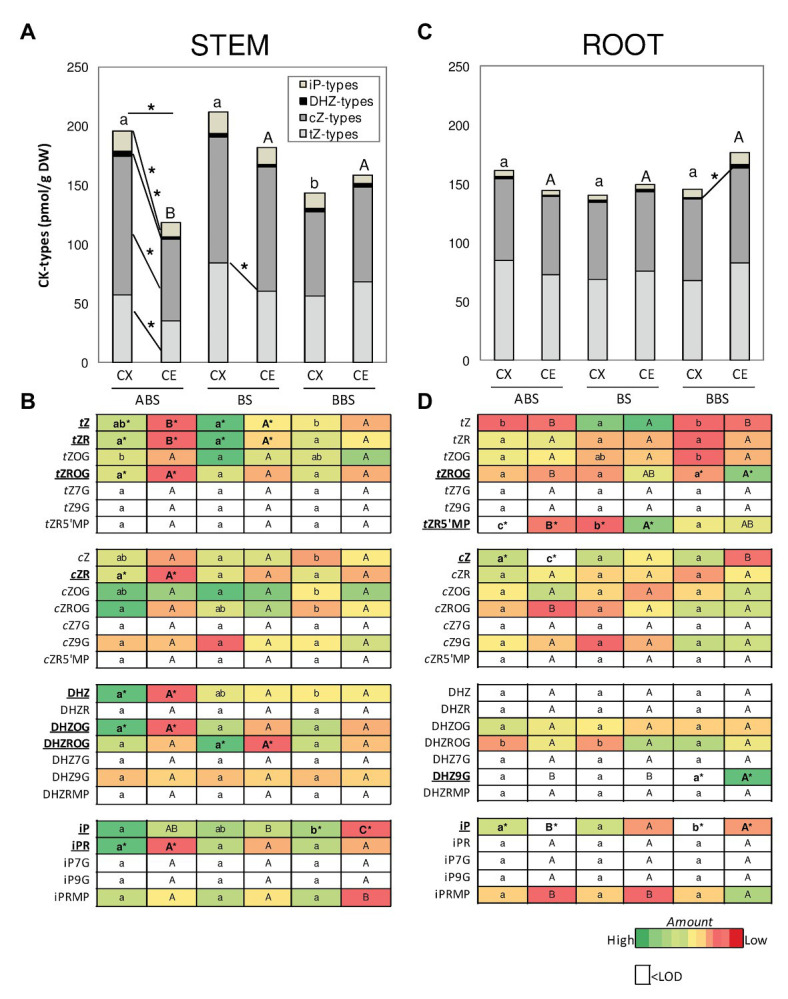
Amounts of different CK-types/forms and conjugates in *P. nigra* bent stem and root. The total amount of *t*Z-, *c*Z-, DHZ-, and iP-types is illustrated in the graph for stem **(A)** and root **(B)** and the corresponding level of each CK-form/conjugate in the heat maps **(C,D)**. Different colors in the heat map indicate the abundance of each CK-form/conjugate in the different samples (*n* = 3): green and red colors indicate, respectively, relative high and low abundance while white color indicates value below the limit of detection (<LOD). Significant differences (*post-hoc* LSD-tests, *p* < 0.05) between the three bent sectors (ABS, BS, and BBS) of the same sides (CX or CE) are indicated, respectively, by lower and capital letter, while those between sides of the same sector are indicated by asterisk. ABS, above bending sector; BS, bending sector; BBS, below bending sector; CX, convex side; CE, concave side.

Hormone profile showed specific distribution patterns of IAA and CKs and related metabolites in the three sectors of two bent organs (stem and root), underlining differences among them and between the convex and concave side of each sector (Figures 4, 5).

In bent stems sectors, the comparison of IAA content showed that in both convex and concave sides, IAA was higher in BS and BBS than ABS and remained unchanged between the two sides of all three bent sectors (Figure 4A, light gray).

IAGlu, in the convex sides, was below the limit of detection (<LOD) in ABS, while increased in BS and BBS (Figure 4B, light gray). In the concave sides, it followed the same IAA trend, but reached the maximum in BBS. Between stem sides, IAGlu was lower in the convex ABS and higher in convex BS compared to the concave opposite side, while no variation was observed between the two sides of BBS.

The IAAsp resulted <LOD in both sides of BS and BBS and high in those of ABS, reaching the maximum in the concave side (Figure 4C, light gray).

The oxIAA did not show any variation among sectors but it was low in all convex side in respect to the concave side (Figure 4D, light gray).

In bent root, IAA amount was lower in the convex BS and BBS sides than opposite concave sides and convex ABS. Furthermore, no significant variations were observed among concave side sectors and within the convex and concave ABS (Figure 4A, dark gray).

The comparison of IAGlu, among sectors, revealed that in convex side was lower in BS and BBS than ABS while in concave side was higher in BS than ABS and unchanged compared to BBS. Furthermore, within sectors, it was lower in the concave ABS and higher in the concave BS compared to the opposite side, while values were comparable in the two sides of BBS (Figure 4B, dark gray).

Analyzing IAAsp amount, among sectors of the convex and concave sides, an opposite gradient was observed. In detail, in the convex side, it was lower BS and BBS than ABS, while in the concave side, it was higher in BBS than ABS and BS. Furthermore, between sides, it was lower on the concave ABS and higher in the concave BBS compared to the opposite sides, while similar values were found between BS sides (Figure 4C, dark gray).

As for the catabolic product oxIAA, in the convex side, it was higher in ABS and BS than BBS, while in the concave side, oxIAA showed the highest value in BS. Between sides, it decreased only in the concave ABS compared to the opposite side (Figure 4D, dark gray).

Distribution of different CKs between sectors and sides of the bent stem and root were analyzed in detail (Figure 5). CKs were specifically categorized according to their side-chain structure into *t*Z-, *c*Z-, DHZ-, and iP-types (Figures 5A,C), specifying the amount of each form/conjugate in the heat maps (Figures 5B,D).

In bent stem, total CKs amount was the highest in ABS and BS of the convex side (Figure 5A) due to the main accumulation of the *t*ZROG, DHZ, and iPR in ABS and the *t*Z, *t*ZR, *c*Z, *c*ZR, *c*ZOG, and *c*ZROG forms/conjugates in ABS and BS (Figure 5B). In the concave side, total CKs was the lowest in ABS, due to a low amount in *t*Z- and *c*Z-type, without any significant differences in the accumulation of specific forms/conjugates (Figures 5A,B).

Between stem sides, *t*Z and *t*ZR were mainly accumulated in the convex ABS and BS with respect to the opposite concave site and of DHZ, DHZOG, *t*ZROG, *c*ZR, and iPR only in convex ABS (Figure 5B). Thus, all CK-types were high in the convex ABS and *t*Z-type in the convex BS (Figure 5A).

In bent root, although total CKs were unchanged between and within sectors, the DHZ-type was slightly accumulated in the concave BBS (Figure 5C). Furthermore, some specific forms/conjugates were differentially accumulated between and within sectors (Figure 5D). In detail, among sectors, in the convex side, high iP amount was found in ABS and BS, and <LOD in BBS. In the concave side, *c*Z and iP were higher in BS and BBS than ABS (<LOD), together the *t*Z and *t*ZR5’MP in BS and the *t*ZROG and DHZ9G in BBS (Figure 5D). Comparing the two sides, the *t*ZR5’MP and the iP resulted <LOD in the convex ABS and BBS, respectively. The iP was <LOD also in the concave ABS together the *c*Z; finally, the *t*ZR5’MP was high in the concave BS and the *t*ZROG and DHZ9G in the concave BBS (Figure 5D).

**Figure 6:**
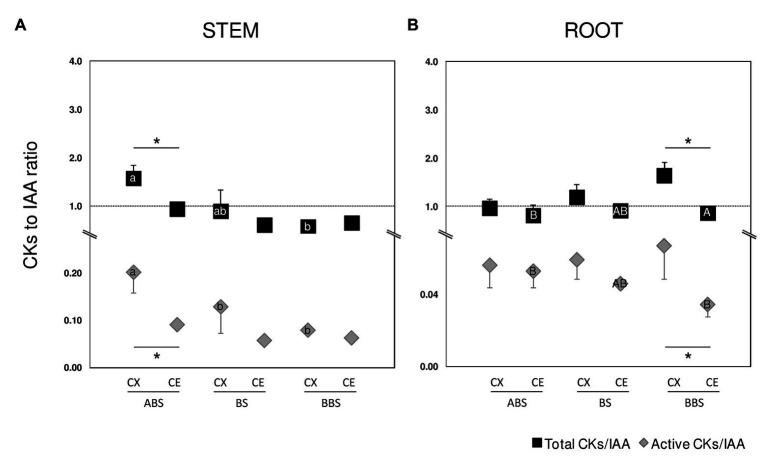
Ratio of total/active CKs to IAA. Stem **(A)** and root **(B)** ratio between the total content of CKs and IAA are indicated by black squared (total CKs/IAA) while ratio between the content of CKs active forms (sum of CK bases and ribosides) and IAA are indicated by gray diamonds (active CKs/IAA). Error bars indicate SD (*n* = 3). Significant differences (Student’s *t* test, *p* < 0.05) between the three bent sectors (ABS, BS, and BBS) along CX and CE sides are indicated by lower and capital letter, respectively, while those between sides of the same sector are indicated by asterisk. ABS, above bending sector; BS, bending sector; BBS, below bending sector; CX, convex side; CE, concave side.

The ratios of total and active CKs to IAA were also calculated (Figure 6) as total, sum of all CK metabolites detected, and active CKs, representing the sum of CK bases and ribosides. In the convex sides of three bent stem sectors, total and active CKs ratios showed the highest value in ABS while no variation was observed in the concave sides. In bent root, BBS showed the maximum value among sectors of the convex sides while no variation was observed between those of the concave sides. Similar trends were shown by both total and active ratios when analyzing differences between sides. In the stem, the content of the CKs and IAA resulted almost the same in both sides of BS and BBS, whereas in the ABS convex, the CKs levels were significantly higher (about 2-fold) compared to IAA (Figure 6A). In root, ABS and BS showed a similar content of two hormones in both root sides, whereas in the BBS concave, the IAA level was significantly higher than to that of CKs, especially considering the CKs active form (Figure 6B).

### Principal Component Analysis of Main Stem and Root Traits According to Bending Sectors

**Figure 7:**
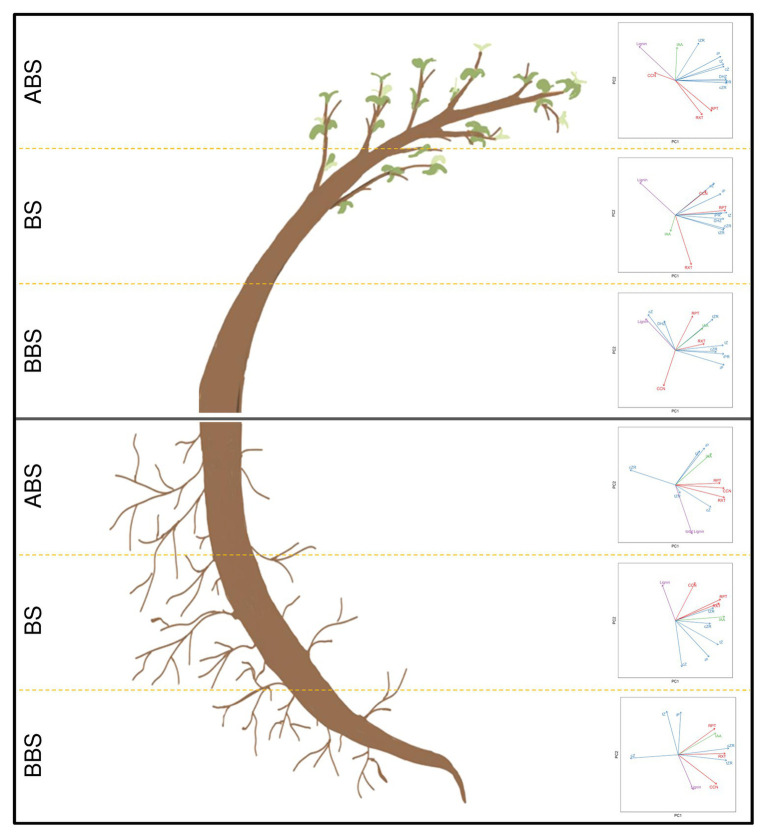
Model summarizing correlation among anatomical, phytohormonal and lignin dataset in bent stem and root. The correlation among main anatomical parameters (CCN, RXT, and RPT – red vectors), phytohormones (IAA – green vectors; CKs free forms – *c*Z, *c*ZR, *t*Z, *t*ZR, iP, iPR, and DHZ – blue vectors) and total lignin content (Lignin – purple vectors) were analyzed by using Principal Component Analysis (PCA). Scatter plots show data variability within each sector (ABS, BS and BBS) of bent stem and root. Data were computed by using FactoMineR package in R and plotted by the two first principal components (PC1 and PC2). Vectors indicate direction and strength of each variable to the overall distribution. CCN, cambial cell number; CKs, cytokinins; *c*Z, *cis*-zeatin; *c*ZR, *cis*-zeatin riboside; DHZ, dihydrozeatin; IAA, indole-3-acetic acid; iP, N-isopentenyladenine; iPR, *N*^6^-isopentenyladenosine; RXT, relative xylem thickness; RPT, relative phloem thickness; *t*Z, *trans*-zeatin; *t*ZR, *trans*-zeatin riboside.

The PCA scatter plots of principle component 1 (PC1) and 2 (PC2) obtained for the three sectors (ABS, BS, and BBS) of bent stem and root are illustrated in Figure 7. From the analysis, in stem, we found a cumulative percentage of PC1 and PC2 accounted for 79.7, 82.5, and 65.2% in ABS, BS, and BBS, respectively. In particular, it was clear that all CKs, IAA, RXT, and RPT were grouped in ABS plot, suggesting that these parameters had a positive correlation among themselves. Also in BS, we observed a good closeness of all the vectors representing CKs and anatomical variables (RXT, RPT, and CCN), meaning their reciprocal high correlation. However, this trend is not emerging for BBS in which a correlation among some CKs (*t*Z, *t*ZR, *c*ZR, iP, and iPR), IAA, RPT, and RXT is clear.

In root, the PC1 and PC2 cumulative percentage accounted for 73.5, 75.2, and 76.9% in ABS, BS, and BBS, respectively. In detail, considering data related to ABS, the scenario seems similar to what is already described for BBS of the stem: anatomical vectors seem to be correlated with IAA and some CKs (*t*Z, *c*Z, and iP) vectors. In the case of BS, we observed that CKs and IAA vectors are next to, and thus correlated to, all anatomical vectors. Finally, in the case of BBS, some CKs (*t*Z, *c*Z, and iP) vectors are spread and not closely related to anatomical vectors that conversely were grouped together with IAA vector.

## Discussion

The modeling of mechanical forces distribution along *P. nigra* L. stem and root axis showed a different intensity of compression and tension forces in the concave and convex sides, respectively, in all the three sectors ABS, BS, and BBS analyzed. However, in bent stem, compression forces increased during time (from t_i_ to t_f_) on concave ABS and BBS, whereas tension forces increased on the convex ABS and BS. Confirming our previous findings (Trupiano et al., 2012b), bent root showed the highest increment of tension forces in the convex side of BS and compression forces in the concave side of ABS. Thus, based on model, in BS, where the maximum of forces intensity was recorded, only the tension forces increased during time in both organs.

Anatomical features and chemical composition variations observed in the convex and concave sides of the three bent woody stem and root sectors were strongly related to both the type of mechanical forces (compression or tension) and the intensity of mechanical force-displacement. In particular in bent stem, compression forces induced the development of secondary phloem on the concave side of BS and BBS, whereas tension forces promoted the formation of RW on the convex ABS and BS, characterized by low vessel number, poor lignifications, high carbohydrate, and G-layer in fiber cell wall, as widely reported in the literature (reviewed in Plomion et al., 2001).

This designed asymmetrical structural organization could represent the best engineering solution to counteract mechanical deformation, and the observed anatomical changes could be important for postural control and to guarantee transport in deforming condition (Mellerowicz and Gorshkova, 2012; Gril et al., 2017).

In bent root, the thicknesses of the cambium and phloem, as well as the area and number of vessel and their wall thickness, were higher in the concave BS and BBS, characterized by the highest magnitude of compression forces compared to ABS. Indeed, compression forces also triggered a significant increment of cambium cell number, xylem, and phloem traits in the concave BS and BBS with respect to the convex side. Thus, according to our previous result (De Zio et al., 2016), the increase of compression forces triggered RW formation, characterized by high relative vessel number and area in the concave BS and BBS of bent root. Moreover, the Py-GC/MS analysis showed high total lignin amount in convex ABS due to high accumulation of S- and G-lignin types, but it did not reveal any significant differences between the two sides in BS and BBS, even though a slight tendency toward higher lignin content was present in the concave sides. All our previous investigation revealed an increase of lignin content in the concave BS and BBS after 6, 12, 13, and 14 months of bending treatment by using Doster and Bostock (1988) method; this data, led us to hypothesize that secondary wood of the concave BS and BBS has characteristic more similar to gymnosperm CW than angiosperm TW (Scippa et al., 2008; Trupiano et al., 2012a, 2014; De Zio et al., 2016, 2019). The disagreement with our previous data could be related to the detection limit of Py-GC/MS technique or duration of the bending treatment, set to 5 months in the preset work. Indeed, evidences showed that Py-GC/MS technique is very accurate in distinguishing lignin-types (H-, G-, and S-type) sub-structures, while it seems inaccurate for the absolute lignin quantification (van Erven et al., 2017). However, it is reasonable to hypothesize that observed differences could be mainly related to the complexity of xylogenesis process influenced by seasonality of annual rhythm (Plomion et al., 2001). Indeed, our previous investigation showed that after 6 months of bending (Scippa et al., 2008; De Zio et al., 2016), lignin slightly increased in the concave BS and BBS, whereas it was strongly accumulated after 12, 13, and 14 months of bending (Trupiano et al., 2012b). Thus, environmental conditions, specially temperature and photoperiod, affecting rate and timing of wood formation (cambium division, cell expansion, followed by the ordered deposition of a thick multilayered secondary cell wall, lignification, and cell death) could regulate the intensity of lignin deposition to determine specific anatomical characteristics of the secondary xylem (Begum et al., 2008; Antonova et al., 2014).

Cambial activity in stem and root resulted differently affected by bending. In detail, in root, the RW formation in the concave BS and BBS was accompanied by an increase of cambial cell number, whereas, in stem, CCN decreased or was unchanged in the convex ABS and BS, respectively. In general, more cambial cells indicate more cambial activity and higher growth rate. The increased growth rate was accompanied by an increase in cambial cell division and, thus, the number of xylem mother cells, able to redesign an anatomical structure optimally tapered for hydraulic purpose (Sorce et al., 2013). Under deforming conditions, as those induced by bending, the control of cambial cells number and their successive differentiation pattern represent important traits to regulate vessel number/size to ensure xylem hydraulic efficiency (Sorce et al., 2013). Therefore, we can hypothesize that the decrease in cambial cell number, observed in the convex ABS stem, could be mainly associated to an increase of cambial cell differentiation rates rather than an absolute decrease in cell division. Furthermore, tension forces, here highly perceived, could control cambial cell division/differentiation rate. Conversely, in root, as reported by Montagnoli et al. (2020), both mechanical force types seem to be equally responsible for the unidirectional RW production toward the concave BS and BBS and in particular, through the compression-related stimulation and tension-related inhibition of cambium activity on their concave and convex sides, respectively.

However, it is well-known that mechanical constraints are the stimuli and that other factors, such as phytohormones, are responsible for controlling the characteristics of either stem or root RW.

The role of auxins in the differentiation of vascular tissue, during both normal development and mechanical constraint is well-documented, although most of the information come from experiments at stem level involving applications of exogenous IAA or IAA-transport inhibitors (Du and Yamamoto, 2007; Tocquard et al., 2014). Despite CKs having a well-established function in cell division, increasing cambium sensitivity toward auxin, and acting as major regulators of wood quality and quantity (Aloni et al., 2006), they have seldom been investigated in relation to RW formation.

A widely accepted model suggests that in the bent stem, the TW forms in the region deficient in IAA, whereas CW is induced by an increase of auxin concentration (for review, see Timell, 1986; Little and Savidge, 1987; Srivastava, 2002). However, Hellgren et al. (2004) demonstrated that TW formation was not linked to any alteration in the balance of endogenous auxin. Moyle et al. (2002) proposed that in poplar stem, initiation of RW after bending stress is caused by an altered auxin sensitivity of specific cells rather than a redistribution of auxin in wood-forming tissues. Moreover, it is widely documented that low IAA concentrations result in slow differentiation, which permits more cell expansion before secondary wall deposition, resulting in wide vessels and a lower vessel density (Bhalerao and Bennett, 2003; Sorce et al., 2013).

In the present study, we found that in poplar bent stems the concentration of the bioactive auxin (IAA) was not linked to any redistribution between the two sides of the three bent sectors, although the lowest amount was found in ABS. It is possible that light stimulus change synergizes with those produced by bending to induce a redistribution of the auxin gradients only in cambial cells. The gradient here hypothesized could be important to enhance firstly cambial cell division and successively their differentiation rate to redirect stem growth orientation upward, against the gravitational pull and towards the light source (Vandenbrink et al., 2014).

In root, as hypothesized in our previous works (De Zio et al., 2016, 2019), the increased IAA level in the concave side could trigger the stress-related anatomical changes in the concave BS and BBS, expressed through the RW formation, due to an increase of cambial activity (Sundberg et al., 2000; Du et al., 2004).

The IAA endogenous levels are tightly controlled through biosynthesis, degradation, transport, and conjugate formation (Casanova-Sáez and Voß, 2019). Interestingly, in this study, IAA metabolites (IAAsp, IAGlu, and oxIAA) closely followed the IAA profile in all sides and sectors of the two bent organs. Despite the functions of IAA, conjugates are still under investigation, it has been proposed that they may serve as storage and protection against IAA oxidative degradation, where an IAA optimum must be guaranteed (Ljung, 2013; Tran and Pal, 2014). Normally, IAA conjugates are present in much lower quantities compared to oxIAA (Pencík et al., 2013; Vayssières et al., 2015); in fact, we noticed, oxIAA > IAGlu > IAAsp concentrations in all three bent stem and root analyzed sectors.

Brunoni et al. (2020) in a feeding experiment using labeled IAA, showed that in Norway spruce, IAAsp was the primary IAA catabolite originating from *de novo synthesis*, highlighting the production of IAAsp as the favorite route for IAA degradation. The group II of GRETCHEN HAGEN 3 (GH3) family of acyl-acid-amido synthetases is demonstrated to be active on IAA amount, playing an important role in catalysis of conjugation reaction in several species and different growth conditions (Ludwig-Müller, 2011). In *Arabidopsis thaliana, iaasp is* a major conjugate, formed by *AtGH3.1–6*, while IAAGlu formation is mediated by *AtGH3.17* (Staswick et al., 2005). In addition, *AtGH3.6*, *AtGH3.5*, and *AtGH3.17* are possible targets of the ARF8 auxin response factor (Tian et al., 2004).

Teichmann et al. (2008) demonstrated that GH3::GUS was strongly induced in response to poplar stem bending, concluding that auxin conjugation is involved in adjusting wood development in response to stress.

According to these evidences, an asymmetrical modulation of specific *GH3* genes in the three bent sectors and sides, that in turn control asymmetrical IAA-amino acid conjugates accumulation, could contribute to the modulation of specific signaling pathways and anatomical alteration we observed in the different sectors of two bent organs.

Depending on how the hormonal response pathways are integrated and on how their biosynthesis and metabolism are related, signals triggered by IAA may be enhanced or dampened, thus yielding additive/synergistic or competitive effects.

Another important hormone group, the CKs, could be a key signal to maintain appropriate levels of auxin biosynthesis (Jones and Ljung, 2011) but they have seldom been investigated in relation to RW formation (De Zio et al., 2019).

Our investigation showed that in poplar bent stems, all CK-types were high in the convex ABS and the *t*Z-type in the convex BS; only in the convex ABS, total and active CKs/IAA ratio was significantly more elevated (about 2-fold) relatively to the opposite concave side. In bent roots, although no variations were observed between different sides and sectors, the total DHZ-type slightly increased in the concave BBS where the CKs/IAA ratio significantly decreased compared to the opposite convex side.

Considering that distinct CKs types/forms are abundant in different sides and sectors of the two organs, each CK type could have specific roles in some important processes, such as cell division/proliferation, cell elongation/differentiation, and xylogenesis (Svacinová et al., 2012). In particular, in poplar bent stem, we may hypothesize that the increase of CKs free bases observed in the convex ABS and BS, together with other forms readily converted to the free base, could have a key role in the control of TW formation, especially in ABS, in which the CKs/IAA ratio was particularly elevated. Indeed, in an *Arabidopsis thaliana* quadruple isopentenyltransferases (*ipts*) mutant, defective for genes encoding free *t*Z and iP biosynthetic enzymes, a loss of vascular cambium activity and a lack of secondary xylem was observed (Matsumoto-Kitano et al., 2008). Also Immanen et al. (2016), exploring the effect of enhanced CKs signaling on vascular architecture, observed a dramatically increased of secondary development in poplar stem.

Conversely, in root, the lack of detection of *t*ZR5’MP in the convex ABS could be consistent with the reduced levels of all *t*Z-type forms. The *t*ZR5MP and *t*ZROG in the concave BS and BBS, respectively, could help to maintain the optimal *t*Z-type levels providing the source for free base conversion (Antoniadi et al., 2015). In *Arabidospis* root, *t*Z regulates the amount of PIN auxin efflux proteins (PIN1, 3 and 7) to create an auxin signaling maximum in protoxylem cells (Bishopp et al., 2011a,b). Auxin here accumulated is in turn able to promote the transcription of AHP6, a negative regulator of cytokinin signaling. The AHP6-mediated inhibition of cytokinin signaling, confines the cytokinin response to the procambial cells, defining vasculature patterning (Mahonen et al., 2006; Bishopp et al., 2011a). Thus, we may suggest that IAA and CKs spatial changes here observed could be important to control specification of vascular pattern (protoxylem identity) also in poplar woody roots.

Finally, the accumulation of DHZ9G form in the concave BBS of bent root, an irreversible *N*-glucosylation inactivation of DHZ (Kieber and Schaller, 2014; Schafer et al., 2015), could be an indication that DHZ-types, compared to the *t*Z-type, might have here a secondary role in RW formation and in gravitropic response (Kollmer et al., 2014). Indeed, here, where also the lowest CK/IAA ratio was found, especially considering the CKs active form, an antagonistic interaction of these two major hormone groups could be proposed to regulate critical aspects of root organogenesis/development and to facilitate downward organ bending and, thus, gravitropic response (Aloni et al., 2006; Waidmann et al., 2019). According to Waidmann and Kleine-Vehn (2020), cytokinins seem to be confirmed a central antigravitropic determinant.

## Conclusion

Static non-destructive mid-term bending triggered the formation of a RW in both *P. nigra* stem and root, each with specific characteristics due to the intensity and type of mechanical forces perceived and related signaling activated in the convex and concave sides of three bent stem and root sectors (ABS, BS, and BBS).

Summarizing all information, in bent stem, the high tension forces in the convex ABS and BS tended to form RW characterized by low vessel number, poor lignifications, high carbohydrate, and G-layer in fiber cell wall. In the bent root, confirming our previous results, the increase of compression forces in the concave BS and BBS triggered RW formation, characterized by high relative vessel number and area. However, here, we did not notice any significant variation in total lignin amount, probably for the duration of bending treatment or the detection technique limit, thus, in this respect, more in-depth analysis is necessary to better assess the observed discrepancy.

These structural organizations may represent the best engineering solution to guarantee postural plant control and water transport in deforming condition and to prepare the bent stem and root to move towards and away, respectively, axial negative-gravitropic growth.

The observed differences between stem and root response to bending highlight how hormonal signaling is highly organ-dependent. Reasonably, the light, gravity and bending signals could synergize in modulating phytohormone gradients in the stem, which is different from that produced by the alone alteration of gravity bending-induced in root. In detail, an antagonistic interaction of CKs and IAA, with opposite trends in bent stem and root, seems to regulate organ-specific response to mechanical constraints. In stem, the CKs free bases could have a key role in the control of unidirectional RW formation, whereas the IAA could be specifically and asymmetrically accumulated only in the cambium zone to induce an earlier and more rapid RW production than in the bent root. Conversely, in root, a key role of IAA in the promotion of cambial cell division and RW initiation was confirmed. Here, a proper active reserve of *t*Z-type could provide the source for the free base conversion important to control vascular cell type identity and development. Thus, CKs are confirmed as central antigravitropic determinant to facilitate upward/downward organ bending in the altered condition of growth orientation.

Further research should be conducted to investigate differences between swayed (dynamic bending) and fixed (static bending) compression and tension forces loading on trees. This information could be critical for understanding how plants maintain/improve their structural integrity in natural mechanical stress conditions (wind, snow, and rain loadings).

## Data Availability Statement

The original contributions presented in the study are included in the article/Supplementary Material, further inquiries can be directed to the corresponding author.

## Author Contributions

GSc and DC conceived the project and provided important insights during the research activities. AM and MT contributed to the experimental system set up and the anatomical analysis. KL supervised and supported the phytohormones experiments. EZ performed hormonal analysis and lignin/carbohydrate determination. MK and IA provided technical and analytical assistance during hormone measurements. GSf modeled the mechanical forces distribution. EZ, DT, MT, and GSf performed the data analysis. EZ, AM, and DT performed data interpretation and manuscript preparation. DT finalized the manuscript. All authors contributed to the article and approved the submitted version.

### Conflict of Interest

The authors declare that the research was conducted in the absence of any commercial or financial relationships that could be construed as a potential conflict of interest.

## Abbreviations

BS: Bending sector
CKs: Cytokinins
CZ: Cambial zone
*c*Z: *cis*-Zeatin
*c*Z7G: *cis*-Zeatin-7-glucoside
*c*Z9G: *cis-*Zeatin-9-glucoside
*c*ZOG: *cis*-Zeatin O-glucoside
*c*ZR: *cis-*Zeatin riboside
*c*ZR5'MP: *cis*-Zeatin riboside-5'-monophosphate
*c*ZROG: *cis*-Zeatin riboside O-glucoside
DHZ: Dihydrozeatin
DHZ7G: Dihydrozeatin-7-glucoside
DHZ9G: Dihydrozeatin-9-glucoside
DHZOG: Dihydrozeatin O-glucoside
DHZR: Dihydrozeatin riboside
DHZRMP: Dihydrozeatin riboside-5'-monophosphate
DHZROG: Dihydrozeatin riboside O-glucoside
IAA: Indole-3-acetic acid
IAAsp: IAA-Aspartate
IAGlu: IAA-Glutamate
iP: N-Isopentenyladenine
iP7G: *N*^6^-Isopentenyladenine-7-glucoside
iP9G: *N*^6^-Isopentenyladenine-9-glucoside
iPR: *N*^6^-Isopentenyladenosine
iPRMP: *N*^6^-Isopentenyladenosine-5'-monophosphate
FW: Flexure wood
oxIAA: 2-Oxindole-3-acetic acid
OW: Opposite wood
PCA: Principal component analysis
RW: Reaction wood
SPE: Solid-phase extraction
*t*Z: *trans*-Zeatin
*t*Z7G: *trans*-Zeatin-7-glucoside
*t*Z9G: *trans*-Zeatin-9-glucoside
*t*ZOG: *trans*-Zeatin O-glucoside
*t*ZR: *trans*-Zeatin riboside
*t*ZR5'MP: *trans*-Zeatin riboside-5'-monophosphate
*t*ZROG: *trans*-Zeatin riboside O-glucoside
