# Supplementary material for: Reaction Wood Anatomical Traits and Hormonal Profiles in Poplar Bent Stem and Root
Source: Front Plant Sci. 2020 Dec 7;11:590985. doi: 10.3389/fpls.2020.590985 (PMC7754185; doi:10.3389/fpls.2020.590985)
Supplement: Supplementary file 1 [file Table_1.doc]

Supplementary Material

**Figure 1S. Anatomical measurements of *Populus nigra* bent stem and root.** Cross-sections of three bent stem and root sectors (ABS, BS and BBS) were divided in four slices by a cross originating in the middle of the primary xylem stele. The reference cross was rotated of 45° in respect of the line (continuous line) dividing the root section in the convex (CX) and concave (CE) sides. Measurements were done in the analyzed area where xylem and phloem were developed after bending application.
